# Blood Counts, Biochemical Parameters, Inflammatory, and Immune Responses in Pigs Infected Experimentally with the African Swine Fever Virus Isolate Pol18_28298_O111

**DOI:** 10.3390/v13030521

**Published:** 2021-03-22

**Authors:** Marek Walczak, Magdalena Wasiak, Katarzyna Dudek, Anna Kycko, Ewelina Szacawa, Małgorzata Olech, Grzegorz Woźniakowski, Anna Szczotka-Bochniarz

**Affiliations:** 1 Department of Swine Diseases, National Veterinary Research Institute, Partyzantów 57 Avenue, 24-100 Pulawy, Poland; grzegorz.wozniakowski@umk.pl (G.W.); anna.szczotka@piwet.pulawy.pl (A.S.-B.); 2 Department of Anatomopathology, National Veterinary Research Institute, Partyzantów 57 Avenue, 24-100 Pulawy, Poland; magdalena.wasiak@piwet.pulawy.pl (M.W.); anna.kycko@piwet.pulawy.pl (A.K.); 3 Department of Cattle and Sheep Diseases, National Veterinary Research Institute, Partyzantów 57 Avenue, 24-100 Pulawy, Poland; katarzyna.dudek@piwet.pulawy.pl (K.D.); ewelina.szacawa@piwet.pulawy.pl (E.S.); 4 Department and Clinic of Animal Internal Diseases, University of Life Sciences in Lublin, Akademicka 13, 20-950 Lublin, Poland; malgorzata.olech@up.lublin.pl; 5 Department of Diagnostics and Clinical Sciences, Faculty of Biological and Veterinary Sciences, Nicolaus Copernicus University in Toruń, Lwowska 1 Street, 87-100 Toruń, Poland

**Keywords:** ASFV, ASF infection, blood counts, biochemical parameters, inflammation, immune response

## Abstract

This study aimed to indicate the influence of infection caused by genotype II African swine fever virus (ASFV)–isolate Pol18_28298_O111, currently circulating in Poland, on blood counts, biochemical parameters, as well as inflammatory and immune responses. Blood and sera collected from 21 domestic pigs infected intranasally with different doses of virulent ASFV were analysed. The infection led to variable changes in blood counts depending on the stage of the disease with a tendency towards leukopenia and thrombocytopenia. The elevated C-reactive protein (CRP) concentrations and microscopic lesions in organs confirmed the development of the inflammation process, which also resulted in an increased level of biochemical markers such as: Aspartate transaminase (AST), creatine kinase (CK), creatinine, and urea. Antibodies could be detected from 9 to 18 days post infection (dpi). Two survivors presented the highest titer of antibodies (>5 log_10_/mL) with a simultaneous increase in the lymphocyte T (CD3+) percentage–revealed by flow cytometry. Results confirmed a progressive inflammatory process occurring during the ASFV infection, which may lead to multiple organs failure and death of the majority of affected animals.

## 1. Introduction

African swine fever (ASF), caused by the African swine fever virus (ASFV), is one of the most dangerous diseases in wild boar and domestic pigs, leading to enormous socio-economic losses in pig farming [1]. As of today, several promising studies have proved the possibility of successful vaccination against ASF. However, there is still no commercially available vaccine against this devastating disease [2,3,4]. Therefore, the prevention of ASF is mainly based on the implementation of effective biosecurity measures in pig farms [5,6]. One of the most important elements in the prevention of the disease is the fast and accurate diagnosis made by veterinary services responsible for the supervision of healthy pigs [7]. Recent studies have shown that the clinical course of the disease caused by genotype II of ASFV, spreading around Europe, may be varied: from acute, causing the animals’ sudden death to chronic or even asymptomatic disease with hardly noticeable, nonspecific clinical symptoms [8,9,10]. Due to this, the diagnosis of ASF in pig farms may be difficult until unexpected and unexplained deaths can be observed. 

The disease was firstly described in 1921 by Montgomery in Kenya, then invaded Europe twice, the first introduction occurred in Portugal (1957) and the second in Georgia (2007) [11,12]. Since then, thanks to the invaluable efforts of many researchers, the pathogenesis of various genotypes of ASFV has been studied and described [13,14,15]. Today, we still see the necessity for investigation of the pathogenesis of the ASFV infection, especially in the scope of genotype II ASFV, recently circulating in Asia and Europe, and the unclearly revealed interactions between the virus and host. The virus enters the host via the pharyngeal mucosa and locates in the nearest lymphatic organs (tonsils and lymph nodes), afterwards spreading to the whole organism via the blood [15]. Macrophages, monocytes, and other cells originating from the myeloid lineage are the target, infected by the pathway of constitutive macropinocytosis and clathrin-mediated endocytosis [16,17]. The infection causes a massive inflammatory reaction which may result in apoptosis in lymphocyte subsets [15,18,19]. The disease also negatively affects blood counts (may lead to lymphopenia, neutropenia, and thrombocytopenia) and impairs the functionality of the internal organs–an elevation of the biochemical parameters–aspartate aminotransferase (AST), lactate dehydrogenase (LDH), and bilirubin have been previously observed [15,20,21]. Moreover, the virus is equipped with a wide range of genes responsible for the evasion of the host’s immune system, that can: modulate interferon response, inhibit apoptosis of target cell, cause MHC Class II antigen processing inhibition, suppress macrophage activation, and induce secretion of immunosuppressive cytokines [19,22,23,24,25]. Altogether, ASF leads mainly to the death of animals, with a small percentage of survivors having no chance of combating the disease effectively. Knowledge about pathology, biological properties, and possible scenarios of the disease’s course plays a crucial role in the early recognition and minimalisation of ASF spread. This indicates the necessity for the biological characterisation of the currently circulating genotype II of ASFV. 

In Poland, ASF was first confirmed in February 2014 [26]. Since then, the disease has been spreading consistently, causing seasonal outbreaks on pig farms. In comparison to Georgia 2007/1 (LR743116) isolate – responsible for the beginning of European epizootics, the Polish isolate – Pol18_28298_O111 contains a few single nucleotide polymorphisms (SNP) in its genome. The genetic variations may be found in the following regions: MGF 110-7L (silent mutation), MGF505-5R (Val->Ile), and K145R (Ser->Tyr). These variations are typical for all the previously described Polish isolates [27]. In addition, the isolate used in this study contains a unique transition within the B475R gene (Glu->Lys) [28]. Our previous study was focused on the clinical characterisation of ASFV genotype II isolate (Pol18_28298_O111) circulating in Poland at that time [9]. The samples collected previously were used in the present study to evaluate the ASFV influence on blood counts, internal organ functionality (based on serum biochemistry), inflammatory response (C-reactive protein and histology), and the immune response of host organisms. 

## 2. Materials and Methods

### 2.1. Virus Properties and Experiment Settings

The Polish strain–Pol18_28298_O111, belonging to genotype II of ASFV, was isolated in 2018 in Western Poland (Lubelskie voivodeship, Chełm district) from the outbreak no. 111. The virus was isolated from the spleen of a lethally affected domestic pig. The virus was propagated and titrated on primary pig alveolar macrophages (PPAM, kindly provided by the Technical University of Denmark) in a RPMI medium (Gibco, Thermo Fisher Scientific, Waltham, USA), supplemented with 10% fetal bovine serum (FBS). 

Twenty-two 5–6-week-old domestic pigs (Danbred Duroc breed) (*Sus scrofa domestica*) were infected intranasally with 1000 haemadsorbing units (HAU) (*n* = 8, Group I–1000 HAU), 500 HAU (*n* = 6, Group II–500 HAU), and 5 HAU (*n* = 8, Group III–5 HAU) of virulent genotype II ASFV field isolate Pol18_28298_O111, as described previously [9]. The control group included six pigs (*n* = 6, Control Group). The pigs were kept in a BSL3-level animal facility at the National Veterinary Research Institute in Puławy, Poland, under conditions described previously [9]. The samples collected from 21 pigs were chosen for further investigation, as described in Section 2.3. The animal trial was approved by the Local Ethical Commission for Animal Experiments in Lublin (approval number 145/2018). All the procedures were conducted according to legal regulations. 

The experimental infection caused by the virus revealed its ability to develop at least three forms of ASF. Among the 22 infected pigs, the acute form of ASF (defined as: fever, 3 to 4 days of life with viremia, a sharp increase of the virus load in the blood) was diagnosed in nine animals, the subacute form of ASF (moderate or high fever, 5–8 days of life with viremia, moderate and a progressive increase of the virus load value in the blood) was diagnosed in 11 animals. Two pigs (#8, #14) survived the infection, presented chronic ASF (varied, clearly visible clinical signs and a constant low virus load in the blood–Figure S1), and were euthanised. Mortality rates varied from 83% to 100%, depending on the infected group. Independently from the infectious dose, in all groups, similar but nonspecific clinical signs were observed [9].

### 2.2. Blood and Blood Counts Analyses

Blood samples were collected at −7, 0, 1, 4 dpi (days post infection), then at least two times a week or daily, whenever clinical signs (i.e., fever) were recorded. Blood was collected into MLVacuCol^®^ tubes (Medlab, Raszyn, Poland), containing anticoagulant (K2-EDTA). The collected samples from 2 days/measurements before and six measurements after the detected viremia were used for the analysis. An EXIGO automatic veterinary blood analyser (Boule Diagnostics AB, Spånga, Sweden) was used to determine the hematological parameters–total white blood cells (WBC), lymphocytes (LYM), granulocytes (GRAN), monocytes (MON), platelets (PLT), red blood cells (RBC), and hemoglobin concentration (HGB). All the results were compared to calibrated EXIGO analyser references for *Sus scrofa domestica.*

### 2.3. Viremia Detection

Viremia was assessed by ASFV DNA detection in the days following the infection, as presented in Figure S1. DNA was extracted from diluted blood samples (1:10 PBS, *v/v*) using a column extraction kit (DNA Mini Kit, Qiagen, Hilden, Germany). The VIROTYPE^®^ ASFV PCR Kit (Qiagen, Hilden, Germany) was used according to the manufacturer’s instructions for viral DNA detection. The qPCR process was conducted in the Rotor-Gene Q thermocycler (QIAGEN, Hilden, Germany). Viremia was not detected in one of the 22 pigs (Group I – 1000 HAU). All the results refer to the 21 infected animals.

### 2.4. Sera and Biochemistry Analyses

The blood samples for sera were collected into MLVacuCol^®^ tubes (Medlab, Raszyn, Poland) containing a coagulation accelerator as well as a separating gel. The blood samples were centrifuged at 1800x g for 10 min at 20 °C, the obtained sera were frozen at −20 °C until further analyses. To determine the influence of the ASFV infection on the internal organ functionality, biochemistry profiles (i.e., hepatic, heart, and kidney) were investigated. The hepatic profile included alanine aminotransferase (ALT), aspartate aminotransferase (AST), γ-glutamyltranspeptidase (GGTP), and albumin levels. Creatine kinase (CK) was assigned to the heart (muscle) profile as well as AST, which is not only specific for hepatocytes, but also among others for the muscle [29,30]. In the kidney profile, creatinine and urea levels were recorded. Biochemistry analyses were done by courtesy of University of Life Sciences, Lublin, Poland, using an ABX PENTRA 400 analyser (Horiba Medical, Montpellier, France). The results were compared with Norwegian crossbred grower pigs as described in reference [29].

### 2.5. C-reactive Protein (CRP) Concentration

The CRP concentration was analysed using a Pig C-reactive protein (CRP) ELISA kit (Life Diagnostics Inc., West Chester, USA). Frozen sera were diluted 2000 times in a dilution buffer and analysed according to the manufacturer’s instructions. Reference intervals were adapted from the manufacturer’s instructions and the available literature [30].

### 2.6. Histology 

Sections of liver, spleen, kidneys, submandibular lymph nodes, lungs, and palatine tonsils were collected and fixed in 10% buffered formalin. Then, the sections were processed in a tissue processor with formalin, water, alcohol, xylene, and paraffin. The paraffin-embedded sections were cooled and cut on a microtome (Microm, Waltham, Massachusetts, USA) into 5 μm thick sections. These sections were stained with hematoxylin and eosin and subjected to the microscopic analysis. The scoring of histopathological lesions was conducted according to the guidelines by Galindo-Cardiel et al. [31].

### 2.7. Antibody Detection and Flow Cytometry

Specific anti-ASFV antibodies were detected by the indirect immunoperoxidase assay (IPT) according to the standard operating procedure (SOP/CISA/ASF/IPT/1), as described by the European Reference Laboratory (EURL) for ASF (CISA-INIA, Valdeolmos, Spain). Antibodies were titrated using IPT in a series of 2-fold dilutions beginning from the 1:40 dilution. 

Sep-Mate™ tubes (Stemcell Technologies, Vancouver, Canada) were applied to obtain peripheral blood mononuclear cells (PBMCs) according to the manufacturer’s instructions. To determine the percentage of T and B lymphocytes, the percentage of cells positively labelled with anti-CD3 and anti-CD21 monoclonal antibodies (Bio-Rad, Hercules, USA) was assessed. 

The evaluation was then performed, where the FSC (Forward Scatter)/SSC (Side scatter) and SSC/CD45 parameters were used to determine the size and the granularity of cells on the basis of lymphocyte gates has been performed. Erythrocytes, platelets, dead cells, and their remains were excluded from the analyses. 

### 2.8. Statistical Analysis

Statistically significant differences between groups were calculated by the one-way analysis of variance (ANOVA) with Dunett’s correction in GraphPad Prism 8.4.2 (GraphPad Software, San Diego, USA). The results are presented as group means with the standard deviation. The statistical significance was defined as the *p* < 0.05 level. 

## 3. Results

### 3.1. Blood Counts 

Varied results regarding white blood cell (WBC) counts were recorded depending on the stage of the disease. Leukocytosis was observed mainly in the first days of the viremia among 11 pigs (6/7 of Group I 1000 HAU, 3/6 of Group II – 500 HAU, and 2/8 of Group III – 5 HAU). The highest leukocytosis was observed in Group I – 1000 HAU (pig #1, 48.7 × 10^9/^L, first day of viremia). During the next few days of viremia, a tendency for the WBC to decrease was observed. Leukopenia was diagnosed in the last days of life among 12 animals (2/7 of Group I – 1000 HAU, 4/6 of Group II – 500 HAU, and 6/8 of Group III – 5 HAU). In the case of four of the infected animals (Group I – 1000 HAU), with previously diagnosed leukocytosis, leukopenia were not identified, however, a tendency towards leukopenia was still noticeable. The difference between maximum and minimum observable WBC counts during viremia was significantly higher in the infected pigs (all experimental groups, *p* < 0.05) in comparison to the Control Group (Figure S2). In parallel, during viremia, granulocytopenia (neutropenia) was diagnosed (at least once) in 10 pigs (1/7 of Group I – 1000 HAU, 4/6 of Group II – 500 HAU, and 5/8 of Group III – 5 HAU). At the same time points, lymphopenia was identified in six pigs (0/7 of Group I – 1000 HAU, 3/6 of Group II – 500 HAU, and 3/8 of Group III – 5 HAU). Monocytosis was noticed in the case of 10 pigs (4/7 of Group I – 1000 HAU, 5/6 of Group II – 500 HAU, and 1/8 of Group III – 5 HAU).

**Figure 1:**
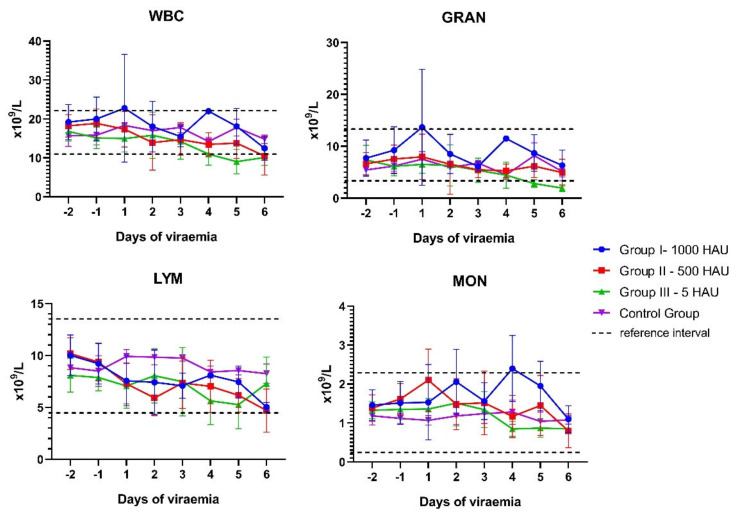
Mean values of white blood cell counts observed during the infection. WBC – white blood cells; GRAN – granulocytes; LYM – lymphocytes; MON – monocytes. Error bars indicate standard deviation.

The analysis conducted in the following 6 days of detected viremia showed the tendency of the total WBC number to decrease (Group II – 500 HAU and Group III – 5 HAU) as well as of granulocytes (Group III – 5 HAU) (Figure 1). 

**Figure 2:**
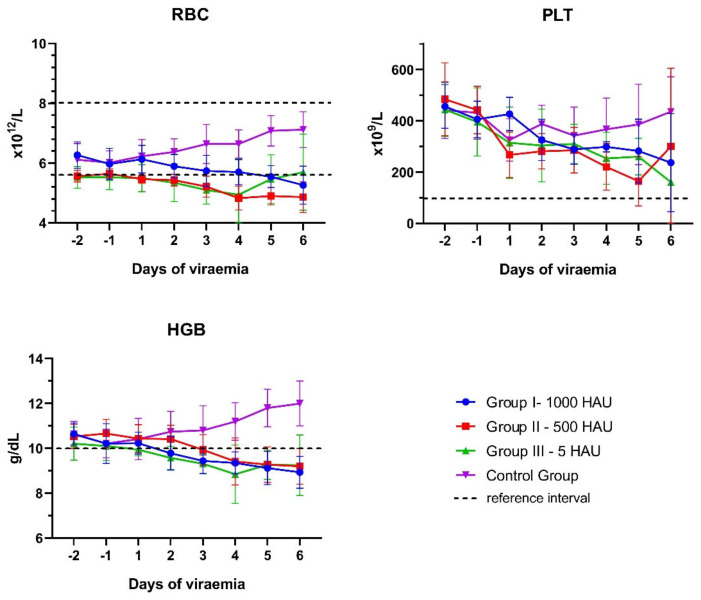
Mean values of red blood cell number (RBC), platelet number (PLT), and hemoglobin concentration (HGB) observed during the African swine fever virus (ASFV) infection. For HGB and PLT, only a low reference threshold is presented. Error bars indicate standard deviation.

The frequency and time of divergence observed in the WBC during the experiment are presented in Table S1. During viremia, a tendency of a decrease in the number of red blood cell (RBC) counts, hemoglobin concentration, and platelet counts was identified (Figure 2).

A slight decrease in the number of erythrocytes (below the reference level) was noticed in the case of 11 out of 21 pigs (2/7 of Group I – 1000 HAU, 5/6 of Group II – 500 HAU, and 4/8 of Group III – 5 HAU), while the concentration of hemoglobin decreased below the reference interval in 95% of the infected animals in the last few days of life (6/7 of Group I – 1000 HAU, 6/6 of Group II – 500 HAU, and 8/8 of Group III – 5 HAU). Only four out of 21 infected pigs presented thrombocytopenia (1/7 of Group I – 1000 HAU, 2/6 of Group II – 500 HAU, and 1/8 of Group III – 5 HAU) (Table S2.).

### 3.2. Biochemical Parameters

**Figure 3:**
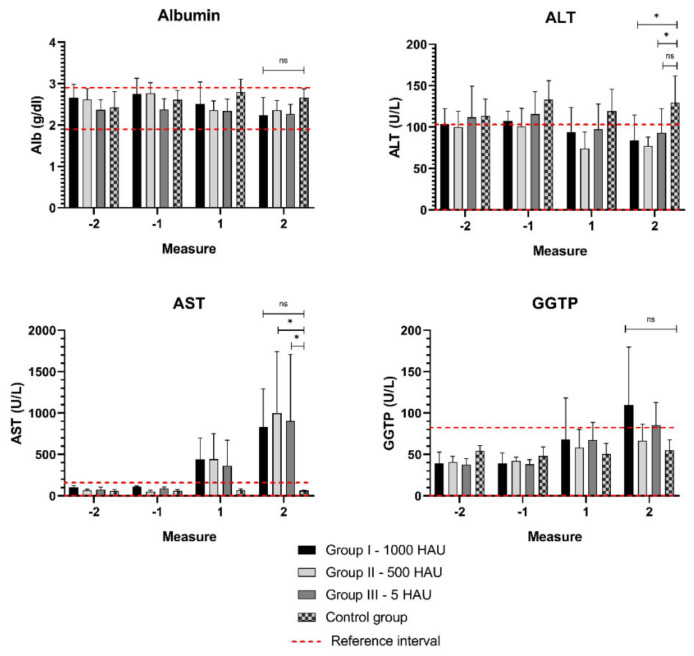
Mean parameters of the hepatic profile. Measurement −1, −2 - two measurements before viremia, measurement 1,2 - the last two days of life with viremia. ALT - alanine aminotransferase; AST - aspartate aminotransferase; GGTP - γ-glutamyltranspeptidase; ns - statistically insignificant; * - statistically significant (*p* = 0.01–0.05). Error bars indicate standard deviation.

A significant increase (*p* < 0.05) in the AST level was recorded in the last day of life in Group II – 500 HAU and Group III – 5 HAU, while no other changes were observed in the hepatic profile (Figure 3). In the case of two animals, AST exceeded the reference level (125 U/L) nearly 20-fold (pig #13, Group II – 500 HAU – 2319 U/L and pig #20 Group III – 5 HAU – 2784 U/L). Surprisingly, in all the groups, the ALT level slightly exceeded the reference interval and was higher in the Control Group than in the infected groups (Figure 3).

**Figure 4:**
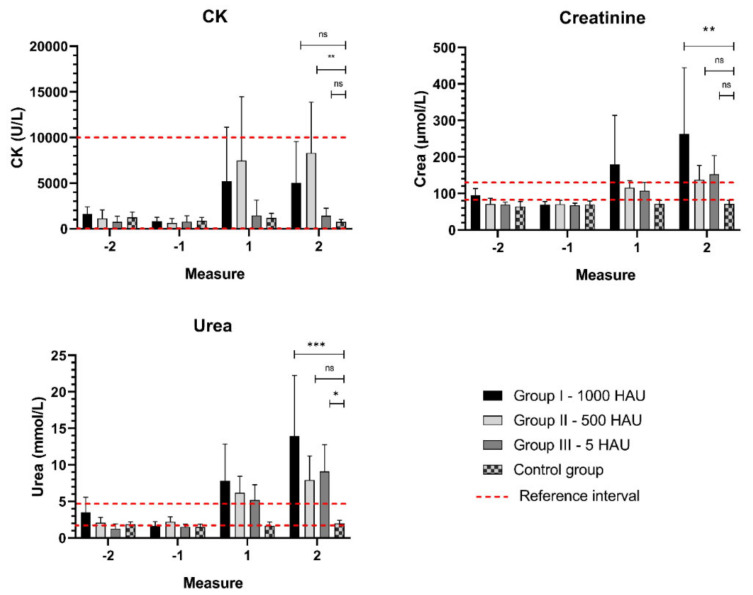
Mean parameters of the kidney profile. Measurements: −1, −2 - two measurements before viremia, 1,2 - the last two days of life with viremia. CK - creatine kinase; ns - statistically insignificant; *,**,*** - statistically significant, respectively; *p* < 0.05, *p* < 0.01, and *p* < 0.001. Error bars indicate standard deviation.

An increased (above the reference) level of CK was recorded in two infected groups of animals (3/8 of Group I–1000 HAU and 3/6 of Group II – 500 HAU) and a statistically significant change (*p* < 0.01) was observed in Group II – 500 HAU. In the kidney profile, significant increases in creatinine (Group I – 1000 HAU, *p* < 0.01) and urea (Group III – 5 HAU, *p* < 0.05 and Group I – 1000 HAU, *p* < 0.001) were recorded, suggesting a failure of the kidneys (Figure 4.).

### 3.3. CRP Concentration and Histology

**Figure 5:**
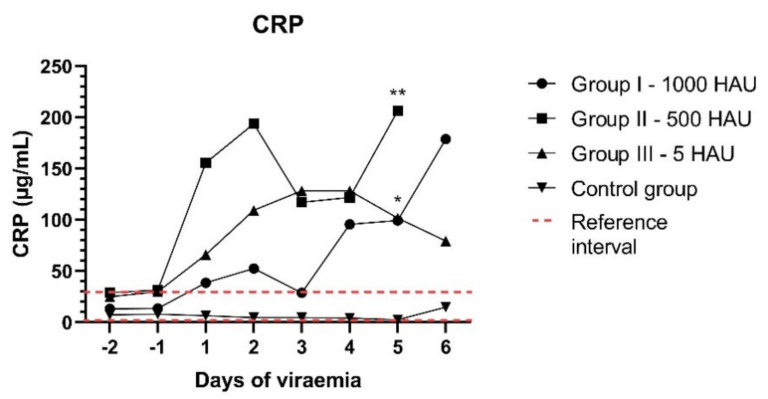
Mean C-reactive protein (CRP) concentrations (μg/mL) in two measurements before (the −2,−1 day) and six measurements after the detected viremia (1–6 days); *,** - statistically significant, respectively; *p* < 0.05, *p* < 0.01.

To determine the inflammatory response, a C-reactive protein (CRP) concentration in the following eight measurements was investigated. The conducted analysis of a mean CRP concentration showed its tendency to increase above the reference level, especially during the last three days of analysis (Figure 5). An increased level of CRP was observed in 18 out of 21 animals (85% - 4/7 of Group I – 1000 HAU, 6/6 of Group II – 500 HAU, and 8/8 of Group III – 5 HAU). The maximum concentration was recorded in Group II – 500 HAU – pig #14 – 564.22 μg/mL and exceeded the reference value (40 μg/mL) approximately 14 times. Statistically significant increases in the CRP concentration were detected on the fifth day of viremia in Group II – 500 HAU (*p* < 0.01) and Group III – 5 HAU (*p* < 0.05). 

**Figure 6:**
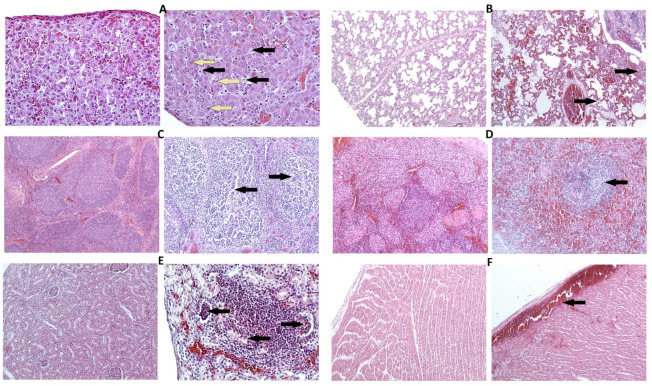
Selected histopathological changes observed in different organs of experimentally infect pigs (**A**–**F** right side) compared to the Control Group (**A**–**F** left side). (**A**) liver (×200), pig #21 (subacute ASF form): Moderate infiltration of hepatic sinusoids with lymphoid cells, with the presence of foamy, degenerated Kupffer cells (black arrows) and scattered necrotic hepatocytes (yellow arrows); (**B**)lung (×200), pig #18 (subacute ASF form): Alveolar and interstitial oedema, congested blood vessels showing moderate vasculopathy, multifocally mild interstitial, and alveolar infiltration with macrophages (arrows); (**C**) palatine tonsil (left ×100, right ×200), pig #2 (acute ASF form): Lymphoid depletion within follicles (arrows); (**D**) spleen (left ×100, right ×200), pig #19 (acute ASF form): Necrosis of lymphocytes within splenic nodule (arrow); (**E**) kidney (left ×100, right ×200), pig #20 (acute ASF form): Focal lymphocytic infiltration in the cortex, necrotic cells in tubular epithelium, and glomeruli (arrows); (**F**) heart (x100), pig #21 (subacute ASF form): Subepicardial hemorrhage.

The histopathological examination of animals associated with the acute and subacute clinical ASF course revealed vascular changes in the lungs, liver, kidneys, spleen, lymph nodes, tonsils, and heart. In the spleen, moderate to severe hyperaemia, vasculopathy, and occasionally haemorrhages were observed. Mild to moderate angiectasia and hyperaemia were observed in the lungs (Figure 6B), in hepatic sinusoids and portal areas, renal cortex and medulla, as well as vessels in lymph nodes and tonsils. In the heart, moderate to massive hyperaemia and subepicardial haemorrhages were found (Figure 6E). Lymphocytic apoptosis and necrosis resulting in lymphoid depletion were visible in follicles in all the tonsils (Figure 6C) and lymph nodes. Multifocal area apoptotic lymphocytes (lymphocytolysis) were observed in the spleen’s white pulp, accompanied by multifocal necrosis of paranodal and reticulo-endothelial areas (Figure 6D). Additionally, in the liver, variable multifocal hepatic necrosis, mild to moderate lymphoid cell infiltrations in periportal areas and sinusoids were found, with an increased number of foamy, enlarged activated or necrotic Kupffer cells (Figure 6A). In lungs, an increased number of activated and necrotic macrophages was found in multifocal interstitial and alveolar areas. In kidneys, multifocally degenerative and occasionally necrotic changes in the tubular epithelium were found next to focal vascular changes, with the presence of necrotic cells in tubules and glomeruli. Occasionally, mild focal lymphocytic infiltrations containing degenerated macrophages were observed (Figure 6F). In chronic cases, mild hyperaemia was found in the examined tissues, more prominent lymphocytic infiltrations were identified in the liver, lungs, and kidneys. The results of histopathological scoring are presented in Table 1.

### 3.4. Immune Response

Among the 21 examined animals, 10 pigs were found ASFV-seropositive, however, only two animals with the chronic form of ASF (#8, #14) presented the highest titer (>5 log_10_/mL) of anti-ASFV antibodies. These pigs survived the infection and were euthanised on the 32nd and 25th days post infection (dpi), respectively. The specific antibodies were detected 2 to 5 days after the detection of viremia (from the 9th to 18th dpi) in sera collected from pigs living ≥4 days with detectable viremia (Table 2).

**Figure 7:**
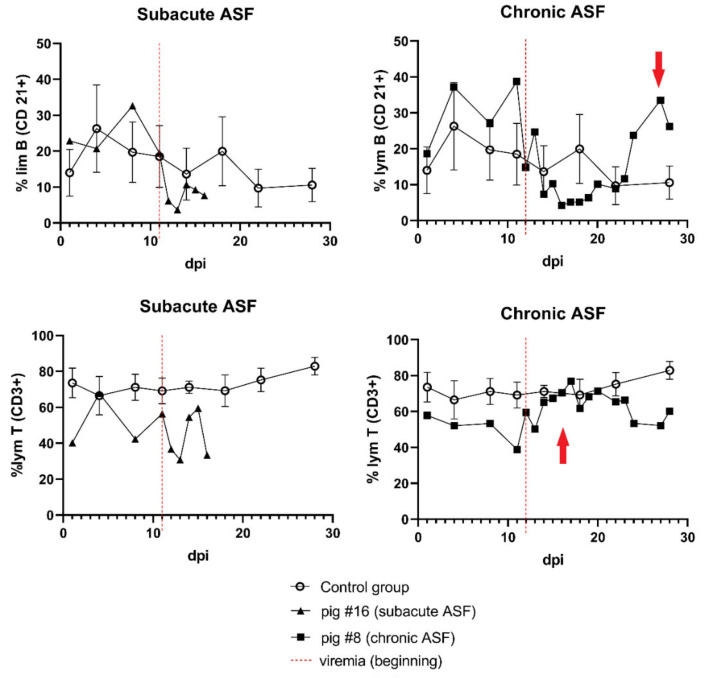
The percentages of the T (CD3+) and B (CD21+) lymphocyte population in pig #8 presenting the chronic ASF form (survivor) in comparison to pig #16 presenting the subacute ASF. The red arrows indicate an increase in both lymphocyte populations. Error bars indicate standard deviation.

Flow cytometry analyses revealed a statistically significant (*p* < 0.01) decrease in the mean percentage of lymphocytes T (CD3+) in one of the infected groups (Group III – 5 HAU) during viremia (Figure S3). At the same time point, there were no statistically significant changes in the mean number of lymphocyte B (CD21+) percentage in all the tested groups, however, in Group III – 5 HAU both populations (CD3+ and CD21+) showed a tendency of lymphocyte B decrease during infection. A comparison of the individual results of pig presenting subacute ASF with survivor pigs presenting chronical ASF showed an increase in the percentage of lymphocyte T (CD3+) shortly after the beginning of viremia, and the next increasing percentage of lymphocyte B (CD21+) (Figure 7). The second survivor (pig #14) presented only an increased percentage of lymphocytes T (CD3+).

## 4. Discussion

ASFV may cause a massive inflammatory reaction due to a release by the host’s organism of a number of proinflammatory cytokines including TNF-α, IL-1α, IL-1β, IL-6, and IL-17, which may result in apoptosis of the affected cells, as well as internal organs failure [15,18,19]. Moreover, it may lead to the occurrence of disseminated intravascular coagulation (DIC)–a pathological process responsible for hemorrhagic lesions [13]. The presented studies confirmed that the infection of pigs by Pol18_28298_O111 isolate leads to inflammation, which may be identified as the high CRP concentration in the blood or by microscopic lesions in affected tissues characterised by interstitial pneumonia, hepatitis, and nephritis accompanied by prominent hyperemia of most of the examined organs. These results are in accordance to those presented by Sanchez-Cordon et al. conducted with the application of genotype I of ASFV [32]. Histopathological changes found in the examined animals were consistent with the inflammation associated with the ASFV infection described previously by other authors [13,33,34]. Vascular changes and an increased number of activated macrophages in the lungs and liver were the most common observations, followed by lymphocytolysis in the spleen, tonsils, and lymph nodes, leading to lymphoid depletion. Consequences of the progressive inflammation process may be observed in both blood counts and serum biochemical parameters – indicators of the health status of the organism. Leukopenia and thrombocytopenia were noted as the most important consequences of infection, although the response to infection may be divergent. Leukopenia (neutropenia, lymphopenia) was described earlier in the case of ASF strain belonging to genotype II [35], however, in the case of genotype X (KWH/12 Kirawira) neutrophilia was observed [36]. Our study also proved a tendency towards leukopenia. The changes were not clearly visible and depended on the stage of infection. In addition, the tendency towards thrombocytopenia has been observed, which according to other studies may be the result of DIC or infection and apoptosis of megakaryocytes – platelet precursors [15]. In the presented study, the Pol18_28298_O111 isolate has slightly affected RBC parameters. The changes recorded in the RBC counts, such as erythrocytopenia or the decrease in hemoglobin concentration, found in this experiment, may partially be a result of organism devastation (i.e., connected to the reduced feed intake) or may be caused by haemolysis, which has been shown previously by Karalyan et al. [15,21]. In agreement with the results obtained by Hühr et al. [37] in this study, we also observed increased monocyte counts in the selected animals. This finding deserves attention, since monocytes are target cells for ASFV and the virus is equipped with genes responsible for antiapoptotic properties [23]. The possibility of probable accumulation of monocytes during the infection needs to be further investigated and elucidated. In contrast to previous papers presented by the Gomez-Villamandos et al. and Zhu et al. in our study, monocytopenia was not observed [13,19].

To determine which internal organs are most affected by ASFV, three biochemistry profiles (i.e., hepatic, cardiac, and kidney) were investigated. The analysis of liver enzymes revealed a significant increase in AST, which is in accordance with the results published by Karalyan et al. and Semeryjan et al. [20,21]. Subsequently, we did not observe an increase in ALT or a decrease in the albumins level, which also supports the results presented by the aforementioned authors [20,21]. These results rather exclude liver failure, especially since AST is not only specific for hepatocytes, but also for myocytes (including cardiomyocytes) [38,39]. The high levels of CK and AST observed in these studies may suggest heart failure, as described previously by Semeryjan et al. [20]. In contrast to Semeryjan et al., a higher CK activity in the investigated sera was identified, which can also be observed in heart failure [40]. In contrast to Semeryjan et al. [20], the third investigated profile (kidney) showed a significant increase in creatinine and urea levels, suggesting kidney failure [41]. These results may be associated with mild necrosis observed in the glomeruli and tubular epithelium. Still, a high probability of other internal organs failure cannot be excluded (i.e., lungs) since the inflammatory processes in the ASF course are generalized and microscopic and macroscopic lesions were visible [9].

Infection caused by a virulent isolate of genotype II ASFV usually occurs with high mortality rates (up to 100%) and a small percentage of survivors [8,9,24]. In these studies, only two pigs (9%) survived the infection. The lack of effective inactivation of the virus by antibodies is frequently associated with a deficiency of neutralizing antibodies [3,42], but the role of anti-ASFV antibodies still remains the subject of discussion [43]. Both survivors presented the highest titers of antibodies, but this may also be a result of a longer exposition to viral antigens. The role of cellular immune response in the effective combat of ASF has been studied before [44,45], however, recent investigations have showed that the solely increased number of cytotoxic cells (T CD8+) may not be sufficient to eliminate the virus [37]. In contrast to the lethally affected animals observed in this study, the survivors presented an increased percentage of lymphocytes T (CD3+) shortly after the beginning of viremia. This suggests that the effective combat of ASF may depend on the simultaneous occurrence of both – the cellular and humoral response of the host organism and is supported by the results of other authors [3,37,44].

## 5. Conclusions

In conclusion, studies conducted on the virulent isolate of genotype II of ASFV (Pol_18_28298_O111) confirmed its ability to develop an inflammation process, which resulted in various changes in blood counts with a tendency towards general leukopenia and depletion of lymphoid tissue. The pathological inflammation could also be seen in microscopic lesions and lead to multiple organs failure with visible biochemical changes in the kidney and heart (muscle) biochemical profiles. The devastating process during the ASF course gives the majority of affected animals no chance to survive the disease. Among the small percentage of survivors, high titers of antibodies with a simultaneous increase in the T-cell percentage could be observed, but the reasons for ASF survival could not be conclusively explained and need to be investigated further.

## Figures and Tables

**Table 1:** Histopathological evaluation of tissues from experimentally infected pigs.

Organ	Histopathological Changes	Score *		
		Acute	Subacute	Chronic
Lung	Oedema	2	2	1
	Congestion/haemorrhage	2	2	1
	Inflammatory infiltrates	1	2	3
Liver	Blood vessels/congestion	2	2	1
	Hepatitis	2	2	1
Kidney	Congestion/haemorrhage (medulla)	2	1	1
	Congestion/haemorrhage (cortex)	2	2	1
	Necrosis	1	1	0
	Renal inflammation	1	1	2
Heart	Congestion/haemorrhage	2	1	0
Spleen	Ratio white pulp/red pulp	3	2	1
	Necrosis	2	1	1
	Lymphocytolysis	2	2	2
	Vascular damage	2	2	2
Tonsil (palatine)	Crypt necrosis	2	2	1
	Lymphocytolysis	2	2	1
	Congestion/haemorrhage	2	2	1
Lymph node (submandibular)	Lymphocytolysis	2	2	1
	Congestion/haemorrhage	2	1	1

* Scoring based on the guidelines published by Galindo-Cardiel et al. [31]. Histopathological changes: (0), Mild (1), moderate (2), severe (3).

**Table 2:** Viremia onset and duration, antibody titres, and detection in infected animals during the experiment.

Group	Pig	1. Day of Detected Viremia (dpi)	Time of Life with Detected Viremia (days)	1. Day of Antibody Detection (dpi)	Average (± SD) Timespan between Detection of Viremia and Antibodies (days)	Maximum Detected Antibody Titre(log10/mL)
Group I 1000 HAU	#1	16	5	n/d	4(± 0.5)	n/d
	#2	4	3	n/d		n/d
	#3	8	3	n/d		n/d
	#4	4	2	n/d		n/d
	#5	n/d	n/d	n/a		n/a
	#6	9	6	n/d		n/d
	#7	10	6	13		4.11
	#8	12	chronic *	16		5.31
Group II 500 HAU	#9	12	4	15	3(± 0.7)	3.51
	#10	11	6	n/d		n/d
	#11	13	6	17		3.51
	#12	5	6	9		3.51
	#13	6	4	9		3.51
	#14	16	chronic *	18		5.01
Group III 5 HAU	#15	9	3	n/d	4(± 1.2)	n/d
	#16	11	7	13		3.81
	#17	11	2	n/d		n/d
	#18	14	5	18		3.20
	#19	11	3	n/d		n/d
	#20	4	4	n/d		n/d
	#21	13	8	18		4.41
	#22	13	3	n/d		n/d

N/d–not detected; n/a–not applicable; *-euthanasia.

## Data Availability

The data presented in this study are available in the present article and in supplementary material.

## Supplementary Materials

The following are available online at https://www.mdpi.com/1999-4915/13/3/521/s1. Figure S1: Viremia detection during the experiment; Figure S2: Differences between maximum and minimum WBC counts observed during the infection (two measurements before, 6 following days of viremia) in experimentally infected pigs in comparison to the Control Group animals; Figure S3: Mean percentages of lymphocytes T (CD3+) and B (CD21+) observed in experimental groups during viremia. Table S1: Frequency and time of observable changes of white blood cell counts identified during the infection; Table S2: Frequency and time of changes observed in the number of erythrocytes, platelets, and haemoglobin concentration.
