# Supplementary material for: Blood Counts, Biochemical Parameters, Inflammatory, and Immune Responses in Pigs Infected Experimentally with the African Swine Fever Virus Isolate Pol18_28298_O111
Source: Viruses. 2021 Mar 22;13(3):521. doi: 10.3390/v13030521 (PMC8004642; doi:10.3390/v13030521)
Supplement: Supplementary file 1 [file viruses-13-00521-s001.zip › Figure S2-.docx]

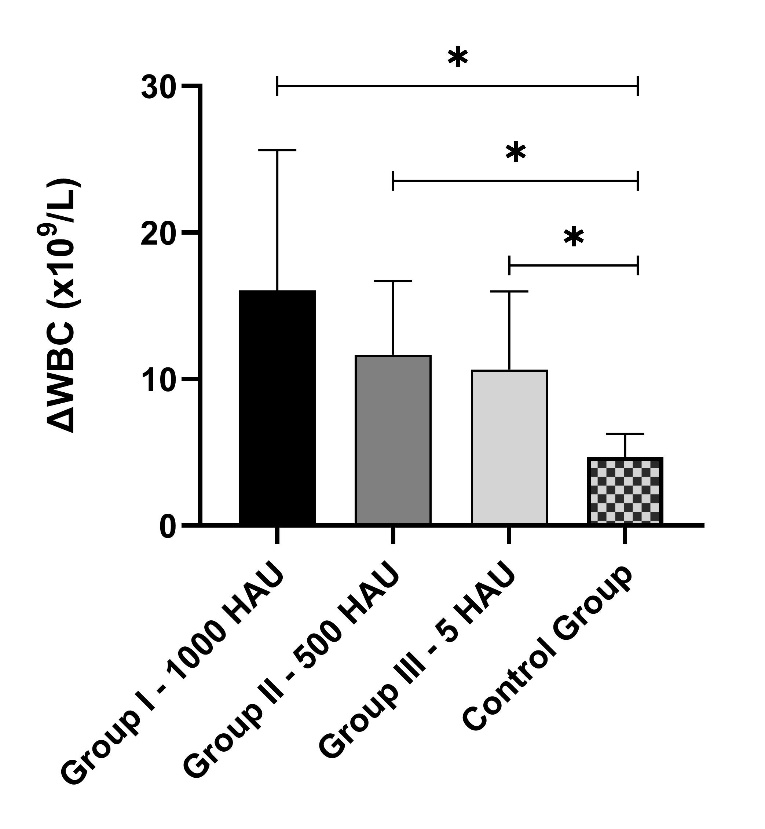


Figure S2. Differences between maximum and minimum WBC counts observed during infection (2 measurement before, 6 following days of viremia) in experimentally infected pigs in comparison to the Control Group animals. *- p<0.05. Error bars indicate standard deviation.
