# Supplementary material for: Blood Counts, Biochemical Parameters, Inflammatory, and Immune Responses in Pigs Infected Experimentally with the African Swine Fever Virus Isolate Pol18_28298_O111
Source: Viruses. 2021 Mar 22;13(3):521. doi: 10.3390/v13030521 (PMC8004642; doi:10.3390/v13030521)
Supplement: Supplementary file 1 [file viruses-13-00521-s001.zip › Table S1 .docx]

Table S1. Frequency and time of observable changes of white blood cell counts identified during the infection. Numbers represent animals with different changes; the number of affected animals to total number of infected pigs is defined as percentage of pigs (%).

| **Type of change** | **Group I**  **(n=7)** | **Group II**  **(n=6)** | **Group III**  **(n=8)** | **%**  **pigs** | **Time of observation** |
| --- | --- | --- | --- | --- | --- |
|  | | | | | |
| **Leukocytosis** | 6 | 3 | 2 | 52% | Before viremia and the first days of viremia |
| **Monocytosis** | 4 | 5 | 1 | 48% | 1 and 2 day of viremia |
| **Granulocytosis** | 3 | 2 | 1 | 29% | Before viremia and the first days of viremia |
| **Lymphocytosis** | 1 | 0 | 1 | 10% | Last days of life |
| **Leukopenia** | 2 | 4 | 6 | 57% | Last days of life |
| **Granulocytopenia** | 1 | 4 | 5 | 48% | Last days of life |
| **Lymphopenia** | 0 | 3 | 3 | 29% | Last days of life |
