# Supplementary material for: Blood Counts, Biochemical Parameters, Inflammatory, and Immune Responses in Pigs Infected Experimentally with the African Swine Fever Virus Isolate Pol18_28298_O111
Source: Viruses. 2021 Mar 22;13(3):521. doi: 10.3390/v13030521 (PMC8004642; doi:10.3390/v13030521)
Supplement: Supplementary file 1 [file viruses-13-00521-s001.zip › Table S2 .docx]

Table S2. Frequency and time of changes observed in the number of erythrocytes, platelets and haemoglobin concentration. Numbers represent animals with different changes; the number of affected animals to total number of infected pigs is defined as percentage of pigs (%).

| **Type of change** | **Group I**  **(n=7)** | **Group II**  **(n=6)** | **Group III**  **(n=8)** | **%**  **pigs** | **Time of observation** |
| --- | --- | --- | --- | --- | --- |
| Erythrocytopenia | 2 | 5 | 4 | 52% | Last days of life |
| Anemia* | 6 | 6 | 8 | 95% | Last days of life |
| Thrombocytopenia | 1 | 2 | 1 | 19% | Last days of life |

*- corresponding to low haemoglobin concentration
